# Improved Air Quality and Asthma Incidence from School Age to Young Adulthood: A Population-based Prospective Cohort Study

**DOI:** 10.1513/AnnalsATS.202402-200OC

**Published:** 2024-10-01

**Authors:** Zhebin Yu, Simon Kebede Merid, Tom Bellander, Anna Bergström, Kristina Eneroth, Anne-Sophie Merritt, Maria Ödling, Inger Kull, Petter Ljungman, Susanna Klevebro, Massimo Stafoggia, Christer Janson, Gang Wang, Göran Pershagen, Erik Melén, Olena Gruzieva

**Affiliations:** ^1^ Institute of Environmental Medicine and; ^2^ Department of Clinical Science and Education, Södersjukhuset, Karolinska Institutet, Stockholm, Sweden;; ^3^ Centre for Occupational and Environmental Medicine, Region Stockholm, Stockholm, Sweden;; ^4^ Stockholms Luft-och Bulleranalys, Environment and Health Administration, Stockholm, Sweden;; ^5^ Sachs’ Children and Youth Hospital, Södersjukhuset, Stockholm, Sweden;; ^6^ Department of Cardiology, Danderyd Hospital, Stockholm, Sweden;; ^7^ Department of Epidemiology, Lazio Regional Health Service/Azienda Sanitaria Locale Roma 1, Rome, Italy;; ^8^ Respiratory, Allergy, and Sleep Research, Department of Medical Sciences, Uppsala University, Uppsala, Sweden; and; ^9^ Institute of Integrated Traditional Chinese and Western Medicine, West China Hospital, Sichuan University, Sichuan, China

**Keywords:** air pollution, asthma, birth cohort

## Abstract

**Rationale:**

The benefits of improved air quality on asthma remain understudied.

**Objectives:**

Our aim was to investigate associations of changes in ambient air pollution with incident asthma from school age until young adulthood in an area with mostly low air pollution levels.

**Methods:**

Participants in the BAMSE (Swedish abbreviation for Children, Allergy, Environment, Stockholm, Epidemiology) birth cohort from Stockholm without asthma before the 8-year follow-up were included (*N* = 2,371). We estimated the association of change in individual-level air pollutant exposure (particulate matter with an aerodynamic diameter ≤ 2.5 μm [PM_2.5_] and ≤ 10 μm [PM_10_], black carbon [BC], and nitrogen oxides [NO_x_]) from the first year of life to the 8-year follow-up with asthma incidence from the 8-year until the 24-year follow-up. Multipollutant trajectories were identified using the group-based multivariate trajectory model. We also used parametric G-computation to quantify the asthma incidence under different hypothetical interventions regarding air pollution levels.

**Results:**

Air pollution levels at residency decreased during the period, with median reductions of 5.6% for PM_2.5_, 3.1% for PM_10_, 5.9% for BC, and 26.8% for NO_x_. A total of 395 incident asthma cases were identified from the 8-year until the 24-year follow-up. The odds ratio for asthma was 0.89 (95% confidence interval [CI], 0.80–0.99) for each interquartile range reduction in PM_2.5_ (equal to 8.1% reduction). Associations appeared less clear for PM_10_, BC, and NO_x_. Five multipollutant trajectories were identified; the largest reduction trajectory displayed the lowest odds of asthma (odds ratio, 0.55; 95% CI, 0.31–0.98) compared with the lowest reduction trajectory. If the PM_2.5_ exposure had not declined up to the 8-year follow-up, the hypothetical asthma incidence was estimated to have been 10.9% higher (95% CI, 0.8–20.8%).

**Conclusions:**

A decrease in PM_2.5_ levels during childhood was associated with a lower risk of incident asthma from school age to young adulthood in an area with relatively low air pollution levels, suggesting broad respiratory health benefits from improved air quality.

Asthma is one of the most common chronic diseases, affecting around 10% of all children and nearly 300 million individuals worldwide (1). Moreover, the prevalence of asthma among children and adolescents is increasing in many regions, including Europe, although a plateau may have been reached in other regions (2, 3). Both genetic and environmental factors contribute to the development of asthma (4, 5). Identifying modifiable environmental risk factors is an important step to reduce the disease burden of asthma.

Ambient air pollution exposure has been consistently associated with increased risk of childhood asthma (6–8), and there is emerging evidence showing that the impact of air pollution exposure during early life on asthma risk exists up to young adulthood (9). Most evidence comes from the extrapolation of exposure–response functions estimated from prospective cohort studies, whereas limited evidence is based on actual reduction in exposure to air pollution. Improvements in children’s respiratory conditions as a result of reductions in air pollution have been reported previously using repeated cross-sectional study design (10–13), but evidence regarding incident asthma so far was only reported by the CHS (Children’s Health Study). The CHS incorporated multilevel design with three successive schoolchild cohorts, allowing for assessment of changing air quality on the consequent asthma incidence at the same age (14). This landmark study with a quasi-experimental design provided important evidence for the substantial health benefits due to the regulatory efforts to improve air quality in the United States, particularly considering reductions from moderately high air pollution levels. Both the European Union and the Swedish air quality guidelines are less stringent than the health-based guidelines proposed by the World Health Organization (WHO) in 2021. In support of implementation of the WHO guidelines, it would be particularly important to shed more light on disease risks at low levels of air pollution (15, 16) and especially on health improvements resulting from reductions in air pollution at such low levels (17).

In the current study, we used the BAMSE (Swedish abbreviation for Children, Allergy, Environment, Stockholm, Epidemiology) cohort in Stockholm County, Sweden, where the air quality is generally good compared with many other cities and regions globally (18), and a general trend of decline in air pollution exposure has been seen in the last two decades (19), with variations in the reduction of air pollution exposure between individuals. We aimed to investigate whether children experiencing larger improvements in air quality would have lower risks of developing asthma from school age until young adulthood.

## Methods

### Study Population and Study Design

We used data from the BAMSE cohort, which is an ongoing population-based cohort with 4,089 newborns recruited between 1994 and 1996 in the Stockholm region, Sweden. Details of the study design and data collection have been published elsewhere (20, 21). Questionnaires were filled in at 2 months after birth (baseline) and 1-, 2-, 4-, 8-, 12-, 16-, and 24-year follow-ups, and clinical examinations were conducted at 4-year, 8-year, 16-year, and 24-year follow-ups (22). To model the air quality improvement, we defined the period from birth to 8-year follow-up as the exposure period and 8-year to 24-year follow-ups as the outcome ascertainment period (*see* Figure E1 in the data supplement). With this study design, we were able to investigate a reasonably long exposure period to observe differences in air quality change between study participants and evaluate incident asthma from school age and above, avoiding the outcome misclassification due to the difficulty in asthma diagnosis for young children. A flowchart of the study design, inclusion, and exclusion are shown in Figure E1. This study was approved by the Swedish Ethical Review Authority (approval 2020-0922), and all participants, or their caregivers during childhood, gave written informed consent.

### Outcome Definition

Asthma was determined based on the definition used in the Mechanisms of the Development of Allergy consortium (23) as the presence of at least two of the following three criteria: *1*) doctor-diagnosed asthma ever; *2*) symptoms of wheeze and/or breathing difficulties in the last 12 months before the date of the questionnaire; or *3*) use of any asthma medication occasionally or regularly in the last 12 months before the date of the questionnaire. Incident asthma is defined as positive if a participant fulfilled the asthma criteria at a given age without having fulfilled the asthma criteria at any previous follow-ups.

### Air Pollution Exposure Assessment

Individual exposure levels to particulate matter with an aerodynamic diameter ≤ 2.5 μm (PM_2.5_), particulate matter with an aerodynamic diameter ≤ 10 μm (PM_10_), black carbon (BC), and nitrogen oxides (NO_x_) from birth to the 24-year follow-up were calculated using a Gaussian dispersion model (24). In brief, local emission inventories for 1990, 1995, 2000, 2011, 2015, and 2020 were used as the input for the dispersion model. For years in between, linear interpolation was used. In addition, a street canyon contribution was calculated for addresses located on busy inner-city streets flanked by contiguous high buildings (until 2012: www.airviro.com/airviro/modules; from 2013 onward: www.au.dk/OSPM). The exposure was calculated at a grid of 35 m for addresses in densely populated areas and 100 or 500 m in less densely populated rural areas. Comparison of the calculated levels with measurements of annual mean values at a traffic monitoring site and two urban background sites (one for BC) resulted in *R*^2^ values of 0.99 for PM_2.5_, 0.99 for PM_10_, 0.98 for BC, and 0.98 for NO_x_ for the period 1990–2020 (2007–2020 for BC because of the availability of monitoring data).

In the current study, the difference in the annual average exposure between the exposure in the first year of life and 8-year follow-up was considered the main index of improved air quality.

### Covariates

Covariates including sex, municipality at birth, parental education, parental occupation, parental asthma or hay fever, and the year the house was built were obtained from the baseline questionnaire. Body mass index was calculated using the height and weight measured during the clinical examination at the 8-year follow-up. Allergic sensitization was defined based on specific IgE levels against common food or airborne allergens in the blood at 8-year, 16-year, and 24-year follow-ups (20). Information on change of residential addresses was obtained from respective questionnaires and supplemented with the Swedish Tax Agency records. Neighborhood-level median income based on the Small Area Market Statistics data (25) from Statistics Sweden was used as the area-level socioeconomic status. Detailed definitions of covariates are presented in the supplementary materials.

### Statistical Analysis

Characteristics of the study population were presented as frequency (proportion) for categorical variables and mean ± standard deviation for continuous variables. Correlations between air pollutants were estimated using the Spearman correlation index. We used the discrete time hazard model (26, 27) to investigate the association of improved air quality with asthma incidence from age 8 until age 24 years. Odds ratios (ORs) were generated as we modeled the conditional probability of developing asthma in each discrete period between follow-ups (27, 28). Models were fitted for each air pollutant separately. Minimal adjustment for covariates determined *a priori* based on a directed acyclic graph (Figure E2) included: age, sex, parental occupation, parental education, area-level socioeconomic status at birth, and baseline air pollution exposure. For the air pollution exposure at baseline, we conducted a principal component analysis and then adjusted for the first principal component (which explained 90% of the variation) in the main model. To account for the spatial clustering effects by municipality, we further assigned random intercept for municipality at birth. Estimates with corresponding 95% confidence intervals (CIs) were presented as per interquartile range (IQR) change.

To investigate the associations of change in multiple air pollutants, as well as fully capture the dynamic change of air pollution, we applied the group-based multivariable trajectory model to identify the multipollutant trajectory groups who shared a similar reduction trend of air pollution exposure. This model assumes a multivariate polynomial regression on time within each group to account for multiple indicators (29, 30). Relative changes of four air pollution levels at 4-year and 8-year follow-ups compared with the exposure levels at the first year of life were used as the input of the group-based multivariable trajectory. Optimal number of the degrees of freedom and number of trajectories were selected based on the Bayesian Information Criteria, posterior probabilities, and sufficient sample size in each trajectory group (31) (supplemental methods). The multipollutant trajectory groups were further modeled as the exposure to investigate the associations with asthma incidence.

We conducted subgroup analyses to investigate whether the association differed by sex, parental asthma or hay fever, maternal smoking during pregnancy, overweight at 8 years (body mass index: 25 kg/m^2^), and residential move after 8-year follow-up via adding the exposure-modifier interaction terms into the model. Asthma subtypes (definition provided in the supplemental methods) were analyzed as secondary outcomes: allergic and nonallergic asthma, obese and lean asthma (32), and asthma with rhinitis and without rhinitis. Several sensitivity analyses were used to test the robustness of the findings: *1*) further adjustment for parental asthma, maternal smoking during pregnancy, and the year the house was built (33); *2*) excluding participants having allergic sensitization before age 8 years; *3*) adjustment for area-level socioeconomic status at the 8-year follow-up; *4*) modification of the exposure calculation period from birth to the 4-year follow-up or to the 12-year follow-up with asthma incidence after respective exposure period as outcome; and *5*) using incident wheezing as the alternative outcome from age 4 years onward.

To provide more intuitive estimates, we applied the G-computation to estimate the potential effects of hypothetical interventions on air pollutants. This approach has often been used to evaluate potential benefit of improving ambient air quality in reducing morbidity and mortality (34–37). The G-computation builds on a regression model of the outcome as a function of exposure and covariates to predict the outcome under counterfactual exposure scenarios (38, 39), which makes it possible to address the question, “what would be the incidence rate of asthma if participants experienced larger or smaller improvement in air quality?” We estimated the asthma incidence under each hypothetical counterfactual scenario and compared that with the natural course (the observed cohort), with 95% CIs generated using 1,000 bootstrap iterations. In the present analysis, we considered the following counterfactual scenarios: *1*) maintaining the air pollution exposure as during the first year of life; *2*) percentage reduction in levels by 10% and 20%; *3*) for PM_2.5_ and PM_10_, we further considered a threshold based on WHO 2021 air quality guidelines (5 μg/m^3^ for PM_2.5_ and 15 μg/m^3^ for PM_10_).

R package “gfoRmula” was used for the G-computation, and steps to perform the analysis have been previously described in detail (40). All analyses were performed using R version 4.0.5, with two-sided *P* values <0.05 considered as statistically significant.

## Results

A total of 2,371 participants were included in the analysis. Distributions of the basic characteristics are presented in Table 1. Comparisons of these characteristics between the included study participants and full cohort showed comparable distributions (Table E1). A total of 395 incident asthma cases (168 male, 227 female) were identified during the period from the 8-year to 24-year follow-ups.

**Table 1.** Distribution of selected characteristics of the study participants

Characteristic	Participant Data
Number of participants	2,371
Incident asthma cases	395
Mean age at onset (range) of asthma, yr	17.24 (10.96–23.87)
Mean age at 8-yr follow-up, yr	7.99 ± 0.49
Sex	
Male	1,165 (49.1)
Female	1,206 (50.9)
Parental education	
Elementary school	48 (2.0)
High school	1,005 (42.4)
University	1,318 (55.6)
Parental occupation	
White collar worker	1,983 (83.6)
Blue collar worker	388 (16.4)
Parental asthma or hay fever	689 (29.1)
Neighborhood median income at birth, SEK	167,000 ± 17,300
Neighborhood median income at 8 yr, SEK	264,000 ± 29,400
Year house was built, at birth	
Before 1940	762 (32.1)
Between 1940 and 1975	991 (41.8)
After 1975	618 (26.1)
Maternal smoking during pregnancy	264 (11.1)
Environmental tobacco smoke at 8 yr	384 (16.2)
Overweight at 8 yr	370 (15.6)
Allergic sensitization to common food or airborne allergens at 8 yr	410 (17.3)
Changed address before 8 yr	1,604 (67.7)

*Definition of abbreviation*: SEK = Swedish Krona.

Data are presented as *n* (%) for categorical variables and mean ± standard deviation for continuous variables, unless mentioned separately.

Individual-level long-term exposure to air pollution decreased moderately from the first year of life to the 8-year follow-up (Table 2). For PM_2.5_, the median (IQR) exposure levels decreased 5.6% (8.0%), with the range from −34.2% to 79.9% (positive value representing air pollution reduction), indicating disparities in the improvement of air quality between the study participants. The median reduction of PM_2.5_ levels was larger for participants who had changed their address before the 8-year follow-up (*n* = 1,604) than for participants who did not move (*n* = 767): 4.5% (5.0%) for nonmovers and 6.7% (13.6%) for movers. Similar trends of decline were observed for PM_10_, BC, and NO_x_, with the largest reduction observed for NO_x_ (median exposure level decreased from 28.9 μg/m^3^ to 16.8 μg/m^3^). High correlations were observed for concurrent air pollution levels.

**Table 2.** Annual average air pollution exposure at the first year of life and 8-year follow-up

	Exposure at the First Year of Life (*μg/m^3^*)	Exposure at the 8-Yr Follow-Up (*μg/m^3^*)	
PM_2.5_	8.79 (1.17)	8.23 (0.92)	
PM_10_	14.4 (3.09)	13.52 (2.47)	
BC	0.96 (0.61)	0.79 (0.45)	
NO_x_	28.90 (24.10)	16.80 (14.03)	

Reduction from the first year of life*		Absolute (*μg/m^3^*)	Relative (*%*)
PM_2.5_	—	0.49 (0.75)	5.61 (8.01)
PM_10_	—	0.43 (1.60)	3.06 (10.56)
BC	—	0.06 (0.28)	5.89 (25.66)
NO_x_	—	8.01 (12.36)	26.81 (25.35)

*Definition of abbreviations*: BC = black carbon; NO_x_ = nitrogen oxides; PM_2.5_ = particulate matter with an aerodynamic diameter ≤ 2.5 μm; PM_10_ = particulate matter with an aerodynamic diameter ≤ 10 μm.

Data are presented as median (interquartile range).

* Absolute air pollution change calculated as levels during the first year of life minus levels at the 8-year follow-up. Relative air pollution change was calculated as absolute change/levels during the first year of life × 100%. Positive values of reduction from the first year of life indicate improved air quality in the later periods.

Reduced levels of PM_2.5_ were associated with decreased risk of incident asthma (Table 3). The OR (95% CI) for asthma incidence was 0.89 (0.80–0.99) per interquartile change in the reduction of PM_2.5_ after adjusting for age, sex, household socioeconomic status, neighborhood-level median income, and air pollution exposure at birth. Trends of decrease in incidence associated with reduction in air pollution were also observed for PM_10_ (OR, 0.93; 95% CI, 0.84–1.03), BC (OR, 0.96; 95% CI, 0.87–1.05), and NO_x _(OR, 0.94; 95% CI, 0.83–1.05). Using relative reduction of air pollution as the exposure retrieved essentially the same results. In the subgroup analyses, no evidence of significant effect modification by parental asthma or hay fever, overweight, maternal smoking during pregnancy, or residential moving was observed (Table E2).

**Table 3.** Associations of improved air quality from birth to 8-year follow-up with asthma incidence from 8- to 24-year follow-up

Air Pollutants	Absolute Reduction	Relative Reduction
PM_2.5_	0.89 (0.80–0.99)	0.89 (0.79–0.99)
PM_10_	0.93 (0.84–1.03)	0.91 (0.82–1.02)
BC	0.96 (0.87–1.05)	0.96 (0.85–1.08)
NO_x_	0.94 (0.83–1.05)	0.97 (0.87–1.09)

*Definition of abbreviations*: BC = black carbon; NO_x_ = nitrogen oxides; PM_2.5_ = particulate matter with an aerodynamic diameter ≤ 2.5 μm; PM_10_ = particulate matter with an aerodynamic diameter ≤ 10 μm.

Data are presented as odds ratio (95% confidence interval). All results were adjusted for age, sex, air pollution exposure at baseline (first principal component of four air pollutants), parental education, parental occupation, and neighborhood median income at birth. Municipality at birth was assigned as random effect. Estimates are presented as per interquartile range (IQR) change of the exposure. IQR for the absolute reduction: 0.75 μg/m^3^ for PM_2.5_, 1.60 μg/m^3^ for PM_10_, 0.28 μg/m^3^ for BC, and 12.36 μg/m^3^ for NO_x_. IQRs for the relative reduction: 8.01% for PM_2.5_, 10.56% for PM_10_, 25.66% for BC, and 25.35% for NO_x_.

**Figure 1.**
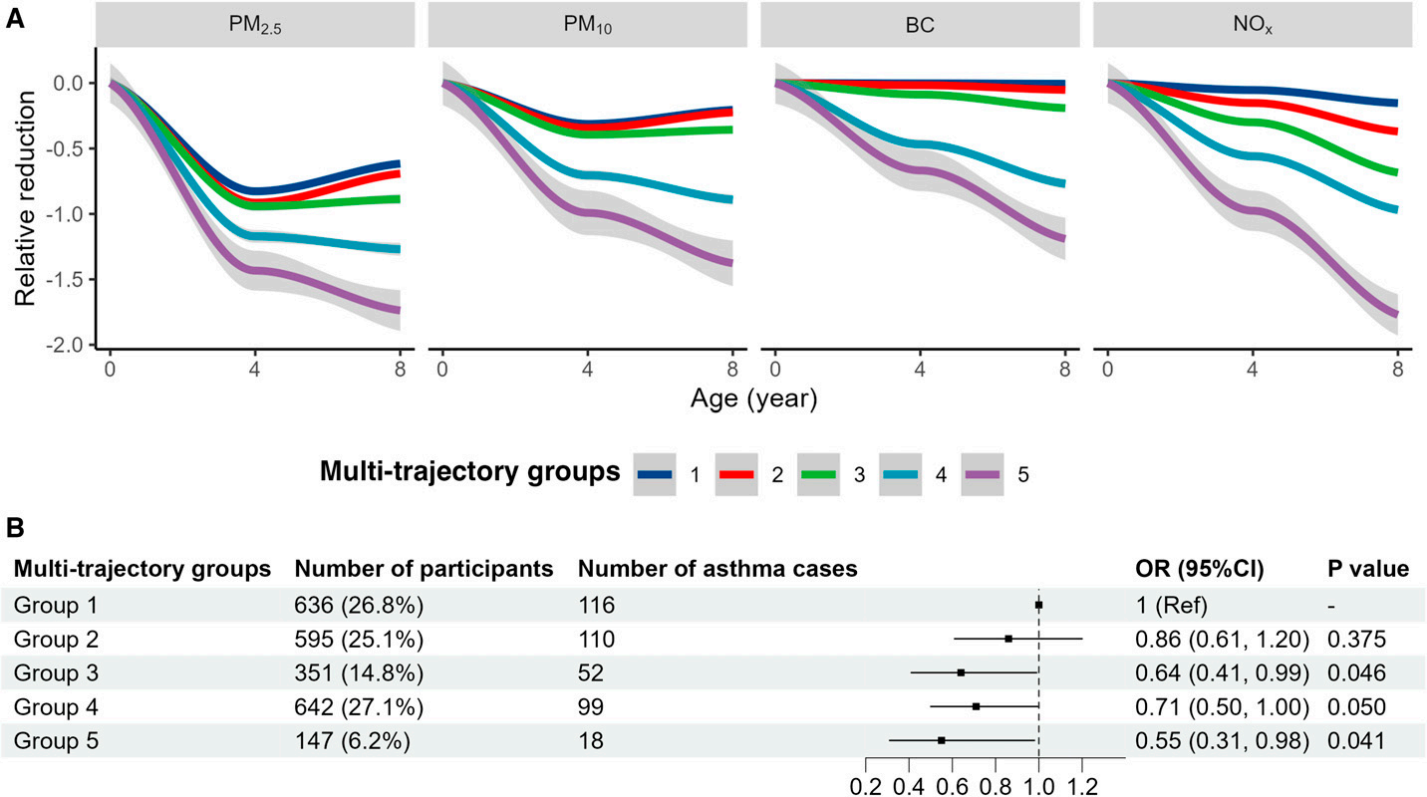
Associations of multipollutant trajectories with asthma incidence from school age until young adulthood. (*A*) Air pollution reduction trend by trajectories identified using group-based multivariate trajectory modeling. The *y*-axis represented the *z*-score of reduction of air pollution: [(exposure level at 4/8) − (exposure level at 0)/standard deviation of exposure level at 0]. (*B*) Percentage reduction of air pollutants within each trajectory and the associations with asthma incidence using the least decline trajectory (trajectory 1) as reference group. Association estimates were adjusted for age, sex, air pollution exposure at baseline (first principal component of four air pollutants), parental occupation, parental education, and neighborhood median income at birth. Municipality at birth was assigned as random effect. CI = confidence interval; OR = odds ratio.

Five multipollutant reduction trajectories from birth to 8-year follow-up were identified (Table E3), with the smallest relative reduction observed for trajectory group 1 and the largest relative reduction observed for trajectory group 5 for all air pollutants. Distribution of basic characteristics by different trajectory groups is presented in Table E4. Trajectories with a larger decline in air pollution were associated with lower risks of developing asthma using trajectory 1 as the reference group after adjusting for the same covariates as in the continuous-exposure models (*P* for trend <0.001), with the most significant reduction in asthma risk observed for trajectory 5 (OR, 0.55; 95% CI, 0.31–0.98) (Figure 1).

The benefits of improving air quality on lower asthma risk were not markedly different in sensitivity analysis, including further adjustment for parental asthma or hay fever, maternal smoking during pregnancy, and the year of building construction (OR, 0.89; 95% CI, 0.79–0.99 for PM_2.5_), neighborhood level median income at 8-year follow-up (OR, 0.84; 95% CI, 0.71–0.99 for PM_2.5_), or when excluding participants who had allergic sensitization at age 8 years (OR, 0.83; 95% CI, 0.72–0.96 for PM_2.5_; Table E5). Association estimates were similar in sensitivity analyses for the multipollutant trajectory analysis (Table E6). The trend of associations still existed when we modified the exposure period to the 4-year or 12-year follow-up (OR, 0.90; 95% CI, 0.77–1.04; and OR, 0.91; 95% CI, 0.80–1.02 for reduction in PM_2.5_, respectively). Protective associations were also observed when using incident wheezing from age 4 years as the outcome (OR, 0.92; 95% CI, 0.85–0.99 for PM_2.5_ and OR, 0.66; 95% CI, 0.45–0.98 for trajectory 5). The protective associations of improved air quality were only seen for nonallergic asthma, whereas no marked difference was observed for comorbidity with obesity or rhinitis (Table E7).

**Figure 2.**
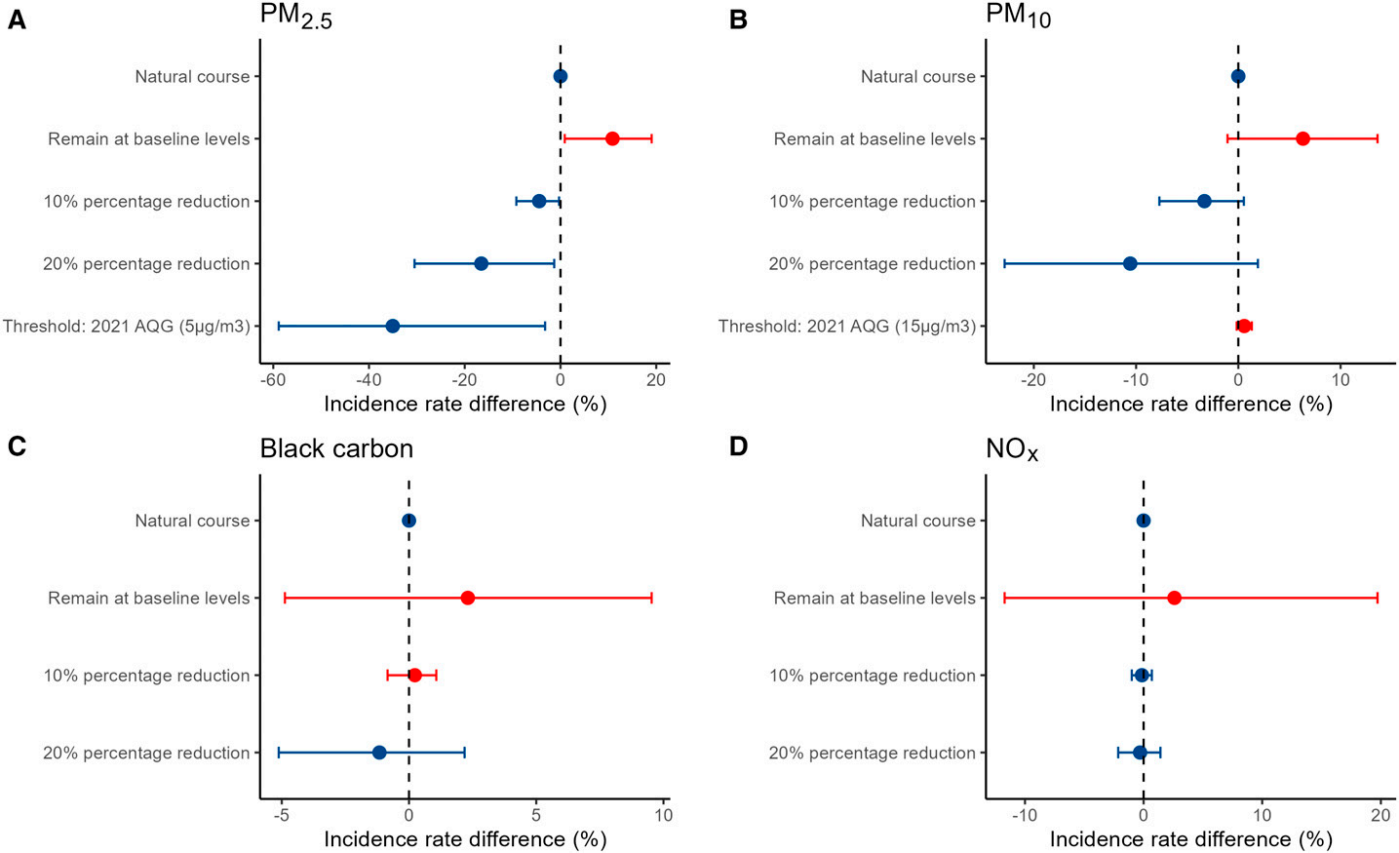
Estimated effects of hypothetical air pollution interventions on asthma incidence. The *y*-axis denotes different hypothetical interventions; the *x*-axis denotes the difference of asthma incidence under certain hypothetical interventions compared with the natural course (no intervention). Blue dots represent reduced asthma incidence under certain intervention, red dots represent increased asthma incidence under certain intervention. AQG = air quality guidelines; NO_x_ = nitrogen oxides; PM_2.5_ = particulate matter with an aerodynamic diameter 92.5 μm; PM_10_ = particulate matter with an aerodynamic diameter 910 μm.

Estimates of the effect of the hypothetical interventions on asthma incidence are displayed in Figure 2. The asthma incidence among our study participants was estimated to be 10.9% higher (95% CI, 0.8–20.8%) if the PM_2.5_ levels remained at their levels in the first year (meaning if the observed decline had not occurred). Had all participants experienced 20% lower PM_2.5_ level at the 8-year follow-up compared with the first year of life, the asthma incidence would have been 16.5% lower (95% CI, 1.3–30.5%) compared with the natural course. The greatest risk reduction for asthma (35.0%; 95% CI, 3.2–58.9%) was estimated if all participants had the PM_2.5_ exposure under the WHO 2021 air quality guidelines (5 μg/m^3^). The effect estimates for hypothetical interventions on PM_10_, BC, and NO_x_ showed similar trends, albeit not significant. Under the multipollutant trajectory scenarios, asthma incidence would have been 14.5% higher (95% CI, −1.6% to 26.0%) if every participant experienced the least decline trajectory and would have been 19.3% lower (95% CI, −36.7% to 1.8%) if all experienced the largest decline trajectory compared with the natural course.

## Discussion

Data from this population-based prospective study showed that sustained improvements in ambient PM_2.5_ levels during childhood were associated with decreases in asthma incidence from school age until young adulthood, after adjusting for the air pollution exposure during the first year of life and socioeconomic status. Multipollutant trajectory analysis identified that participants who experienced the largest decline of four air pollutants had substantially lower risk of developing asthma compared with the trajectory of least decline in air pollutants. Moreover, we estimated that the asthma incidence would have been higher if PM_2.5_ had not declined during the 1994–2002 period and would have been markedly lower if more improvements in clean air had been achieved. There was no evidence that the protective associations of improved air quality were modified by sex, parental asthma or hay fever, overweight, maternal smoking, or residential moving.

To our knowledge, only the CHS (14) reported that decreases in ambient nitrogen dioxide and PM_2.5_ over two decades were associated with lower asthma incidence during the 4^th^- to 12^th^-grade period (previous findings on reduced air pollution exposure and respiratory symptoms are summarized in Table E8). Our findings regarding the associations for PM_2.5_ concur with the results from the CHS, although estimates cannot be directly compared because of differences in study design and air pollution exposure assessment. It should be noted that the estimated exposure difference in the CHS was based on the change of regional (community level) air pollution levels with moderate exposure levels (baseline PM_2.5_ level range from 21.3 to 31.5 μg/m^3^), whereas the current study used a validated dispersion model to estimate levels at the home address with a higher spatial resolution and in a much lower exposure level setting (baseline PM_2.5_ level, 8.8 μg/m^3^). Furthermore, by including the data at 16-year and 24-year follow-ups, our results added to the evidence that benefits of improved air quality on respiratory conditions existed from adolescence to young adulthood, which previously were only shown in children (11–14) or adults (41) (Table E8).

We observed that a moderate decrease in air pollution exposure from the baseline to the 8-year follow-up was associated with decreased risk of developing asthma onward until young adulthood. Similar but attenuated associations estimates were observed when we modified the design by defining the baseline to 4-year follow-up as the exposure period. This could be partially explained by a need for sufficient decreases in air pollution exposure to observe the related health effects, which we may not have achieved by only looking at exposure change from 0 to 4 years. Also, by studying school age and above, we believe we reduced the risk of including wheezing cases (rather than asthma). The strongest associations were indicated for PM_2.5_ compared with PM_10_, BC, and NO_x_. We observed similar stronger associations for PM_2.5_ with other health outcomes, including lung function growth, cognition, and post–coronavirus disease (COVID-19) conditions (19, 42, 43) than those for other pollutants. Road traffic exhaust, particles from road wear, and residential wood burning were the dominant local emission sources for PM_2.5_, whereas exhaust emissions from road traffic were the main source for BC and NO_x_ (42), indicating that apart from traffic-related emissions, other emission sources also play a role in the health effects of PM_2.5_ exposure. Changes in the chemical composition of PM_2.5_ or in the relation between NO_x_ and other combustion-related pollutants, interaction with meteorological factors, and differences in exposure variability may also contribute to the differences of the estimates, which warrants future investigation.

Our findings have important public health implications. Despite the comparatively low levels of air pollution in Sweden, we still observed improvements in asthma risk associated with reduced air pollution. Our findings align with other studies that have explored the health benefits of reducing air pollution in low-exposure settings. A nationwide study in Canada, where the average PM_2.5_ level was approximately 10 μg/m^3^, revealed that lowering PM_2.5_ exposure through residential relocation was linked to reduced mortality (44). We also reported that decreasing air pollution levels were linked to improved lung function growth in the same population (19). Research behind several studies on health effects related to low-level air pollution exposure similarly emphasized the evident risks of mortality and disease in areas with low air pollution levels (45–47). Based on the G-computation, we estimated that the reduction in air pollution during the 1994–2002 period prevented approximately 10.9% of new asthma cases (roughly equivalent to 341 asthma cases per 100,000 person-years [48]). However, these estimates will require validation in future health impact assessment studies. Furthermore, the fact that asthma is documented risk factor for chronic respiratory disease in later life (49), as well as the substantial cost both directly (health care) and indirectly (such as school absenteeism, sick leave, and reduced working capacity) related to asthma, highlight the potential long-term benefits from mitigating ambient air pollution.

The stronger associations with improved air quality observed for nonallergic asthma (compared with allergic asthma) concurred with the so far relatively limited evidence that distinguishes allergic and nonallergic asthma (27, 33, 50) and also the lack of consistent associations found for overall IgE levels with air pollution (51). One potential explanation might be that air pollution exposure mainly triggers increased levels of nonallergic inflammation (52). It is also possible that allergic asthma is mainly determined by the allergen with a stronger genetic predisposition, which may be less influenced by the change of air quality. The sample size limited the statistical power to shed more light on other asthma subtypes (such as eosinophilic asthma or type 2 asthma), which remain to be addressed by future studies, ideally from collaboration of multicohorts.

### Strengths and Limitations

The strengths of the current study were the use of high-resolution spatiotemporal air pollution modeling to calculate the individual exposure, benefit estimates based on the within-cohort comparison, as well as the availability of potential confounders, which together reduce the likelihood of confounding arising from between-cohort comparisons in previous studies (53). Moreover, we used a causal inference modeling framework to estimate the asthma incidence under several hypothetical air pollution scenarios, which may improve the translation of the results to policymakers (54).

We also acknowledge several limitations of the current study. One potential limitation is regression to the mean due to the changes in air pollution that were measured based on two time points (exposure in the first year of life minus the exposure at 8-year follow-up), although the protective associations were further confirmed by the exposure trajectory analysis, which used exposure at baseline and 4-year and 8-year follow-ups. We were unable to explore the prenatal exposure because of lack of data. The relatively small number of incident asthma cases also limited exploration of a longer exposure period with incident cases at higher ages. Selection bias may be of concern, but the background characteristics of the included participants and the full cohort tend to be comparable. We cannot completely rule out the existence of unmeasured confounding factors that may covary with air pollution reduction and have associations with the risk of asthma. Measurement error in the air pollution exposure assessment may also influence the observed results; for example, we lack the data on means of commuting to school and time or location for outdoor leisure activities. The imprecision in estimates resulting from measurement error in exposure generally leads to reduced power of the study to detect associations (55). Because of high correlations between the air pollutants, we were unable to disentangle the independent effect of specific pollutants; instead, we used multipollutant trajectories and found that benefits of experiencing large reductions in all pollutants were substantial. We believe this has important public health implications, as regulation efforts to mitigate climate change (56), such as reducing anthropogenic emissions from combustion of fossil fuels, are likely to reduce the levels of air pollutants, which share similar emission sources. Other air pollutants, such as ozone and ultrafine particles, may also be relevant, which needs to be investigated in the future. Several assumptions should also be noted when interpreting the results of G-computation: we assumed exchangeability (no unmeasured confounding factors), consistency (exposure levels correspond to a well-defined intervention), and positivity (all exposure values are experienced in every confounder subgroup).

### Conclusions

Our results indicate that a decrease in ambient PM_2.5_ levels during childhood was associated with a lower risk of incident asthma from school age to young adulthood.
